# Rhizosphere Bacteria in Plant Growth Promotion, Biocontrol, and Bioremediation of Contaminated Sites: A Comprehensive Review of Effects and Mechanisms

**DOI:** 10.3390/ijms221910529

**Published:** 2021-09-29

**Authors:** Qudsia Saeed, Wang Xiukang, Fasih Ullah Haider, Jiří Kučerik, Muhammad Zahid Mumtaz, Jiri Holatko, Munaza Naseem, Antonin Kintl, Mukkaram Ejaz, Muhammad Naveed, Martin Brtnicky, Adnan Mustafa

**Affiliations:** 1 College of Natural Resources and Environment, Northwest Agriculture and Forestry University, Yangling 712100, China; syedaqudsia.saeed@yahoo.com; 2 College of Life Sciences, Yan’an University, Yan’an 716000, China; 3 College of Resources and Environmental Sciences, Gansu Agricultural University, Lanzhou 730070, China; fasihullahhaider281@gmail.com; 4 Institute of Chemistry and Technology of Environmental Protection, Faculty of Chemistry, Brno University of Technology, Purkynova 118, 612 00 Brno, Czech Republic; kucerik@fch.vut.cz (J.K.); martin.brtnicky@seznam.cz (M.B.); 5 Institute of Molecular Biology and Biotechnology, The University of Lahore, Defense Road, Lahore 54000, Pakistan; zahidses@gmail.com; 6 Department of Agrochemistry, Soil Science, Microbiology and Plant Nutrition, Faculty of AgriSciences, Mendel University in Brno, Zemedelska 1, 613 00 Brno, Czech Republic; jiri.holatko@mendelu.cz (J.H.); kintl@vupt.cz (A.K.); 7 Institute of Soil and Environmental Science, University of Agriculture Faisalabad, Faisalabad 38040, Pakistan; munaza127@yahoo.com (M.N.); muhammad.naveed@uaf.edu.pk (M.N.); 8 Agricultural Research, Ltd., Zahradni 400/1, 664 41 Troubsko, Czech Republic; 9 School of Environmental and Municipal Engineering, Lanzhou Jiaotong University, Lanzhou 730070, China; mjmukkaram@gmail.com; 10 Biology Center CAS, SoWa RI, Na Sadkach 7, 370 05 České Budějovice, Czech Republic

**Keywords:** rhizobacteria, plant growth, abiotic stresses, sustainable agriculture, plant–microbe interactions

## Abstract

Agriculture in the 21st century is facing multiple challenges, such as those related to soil fertility, climatic fluctuations, environmental degradation, urbanization, and the increase in food demand for the increasing world population. In the meanwhile, the scientific community is facing key challenges in increasing crop production from the existing land base. In this regard, traditional farming has witnessed enhanced per acre crop yields due to irregular and injudicious use of agrochemicals, including pesticides and synthetic fertilizers, but at a substantial environmental cost. Another major concern in modern agriculture is that crop pests are developing pesticide resistance. Therefore, the future of sustainable crop production requires the use of alternative strategies that can enhance crop yields in an environmentally sound manner. The application of rhizobacteria, specifically, plant growth-promoting rhizobacteria (PGPR), as an alternative to chemical pesticides has gained much attention from the scientific community. These rhizobacteria harbor a number of mechanisms through which they promote plant growth, control plant pests, and induce resistance to various abiotic stresses. This review presents a comprehensive overview of the mechanisms of rhizobacteria involved in plant growth promotion, biocontrol of pests, and bioremediation of contaminated soils. It also focuses on the effects of PGPR inoculation on plant growth survival under environmental stress. Furthermore, the pros and cons of rhizobacterial application along with future directions for the sustainable use of rhizobacteria in agriculture are discussed in depth.

## 1. Introduction

Recently, crop production has been facing serious threats due to various biotic and abiotic stresses. Feeding the growing population and enhancing agricultural production on limited land are significant challenges for researchers and farmers in the current era [1]. In addition, present agricultural practices, such as the use of fertilizers, pesticides, herbicides, and irrigation with untreated wastewater, pose serious threats to the environment and cause soil degradation [2]. Moreover, urbanization and industrialization have caused a significant reduction in the agricultural area, which has further motivated scientists to develop sustainable strategies to increase crop yields from the already shrinking cropped area [3]. Increasing the area available for crop production has proven difficult; therefore, strategies should be developed to increase the crop yield per unit area in a way that prevents the further degradation of natural resources [4]. Therefore, adopting alternative approaches in today’s agriculture is necessary for ensuring environmental sustainability and future food security. 

Several technical strategies suggested in the past involve enhancing agricultural production by reducing agricultural inputs such as fertilizers and pesticides. In this context, the use of rhizobacteria has been increasingly gaining momentum. Rhizobacteria reside in the rhizosphere, and those having beneficial effects on plants are termed plant growth-promoting rhizobacteria [5,6]. These rhizobacteria are equipped with a number of mechanisms (both direct and indirect) through which they improve plant growth in diverse agricultural settings. Several previous studies have reported the natural enhancement of plant growth of field crops by applying plant growth-promoting rhizobacteria (PGPR). The mechanisms for plant growth promotion used by rhizobacteria, which inhabit the rhizosphere, include metabolic adjustments, adjustments in phytohormone levels, production of exopolysaccharides, root colonization, and enhancement of nutrient availability [7,8,9]. These rhizobacteria also indirectly improve plant growth by inducing plant resistance to various biotic and abiotic stresses, such as pathogen attack and heavy metal contamination, using such mechanisms as the production of antibiotics, induction of induced systemic resistance, rhizosphere competence, and production of antagonistic substances for biocontrol [7,10,11,12,13]. Moreover, the mechanisms used by rhizobacteria for the bioremediation of contaminated soils include the production of biosurfactants and siderophores, biosorption, ACC deaminase activity, and production of polymeric substances [14,15,16].

**Figure 1:**
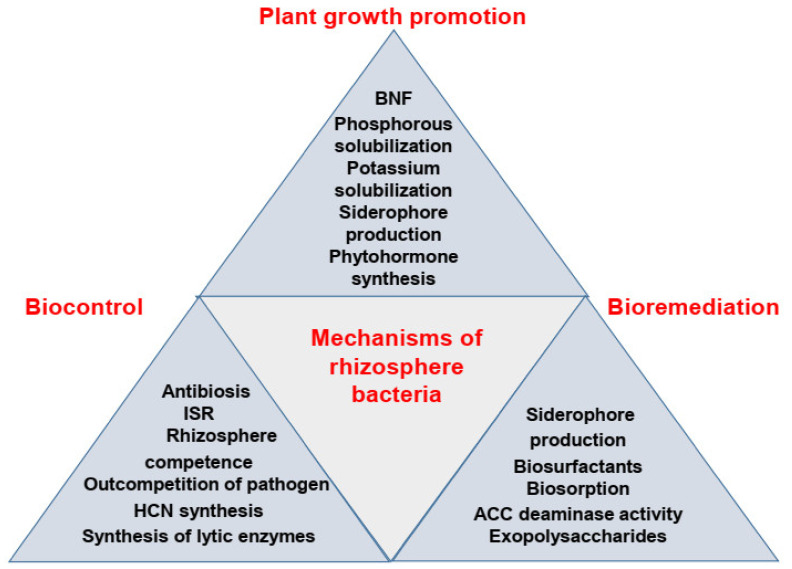
An overview of the mechanisms employed by rhizobacteria for plant growth promotion, biocontrol of plant pests, and bioremediation of contaminated soils. BNF, biological nitrogen fixation; ISR, induced systemic resistance; HC, hydrogen cyanide; ACC, 1-aminocyclopropane, 1-carboxylic acid.

In this review, we summarize the key knowledge of plant growth promotion resulting from rhizosphere bacterial application in diverse agricultural settings. Here, we synthesize the main findings and highlight the in-depth analysis of mechanisms used by rhizobacteria for plant growth promotion, biocontrol, and bioremediation of contaminated sites (Figure 1) in a comprehensive manner. We then discuss the pros and cons of rhizobacterial application in modern agriculture for improving plant growth, and we then evaluate technical suggestions for the future use of rhizobacteria in agriculture. 

## 2. Plant Growth Promotion by Plant Growth-Promoting Rhizobacteria (PGPR): An Overview

During their development, plants are in intimate and continuous contact with microorganisms present in the root vicinity, known as the rhizosphere. Microbes living in the rhizosphere of several plants and having several positive effects on the host plant through various mechanisms are usually termed plant growth-promoting rhizobacteria (PGPR) [17,18]. In the rhizosphere, plant roots secrete a number of exudates that act as attractants for microbes, which eventually improve the physicochemical properties of the surrounding soil. On the other hand, these exudates maintain the function and structure of microbial communities near plant roots [9,19]. Plants and bacteria form symbiotic associations to alleviate abiotic stresses [20,21,22,23]. PGPR can assist plants in their growth by fixation of atmospheric nitrogen, producing siderophores, generating phytohormones (auxins, gibberellins, cytokinins), solubilizing phosphorus (P), or synthesizing stress-relieving enzymes [24]. Moreover, certain bacteria improve the accessibility of essential nutrients, improve root progression, and lessen stress-induced damage by modifying plant defense systems [25,26]. Furthermore, PGPR indirectly help plant symbionts by initiating induced systemic resistance, exerting an antibiosis effect, and potentially improving the content of plant cell metabolites [25,27]. PGPR can withstand hostile natural conditions such as shortage of water, salt stress, weed invasion, lack of nutrients, and heavy metal pollution [28]. The use of PGPR could help to enhance and improve sustainable agriculture and natural stability. These PGPR can be found in association with roots (in the rhizosphere), which enhance plant growth in the absence of pathogens or lessen the harmful effects of pathogens on crop yield by antibiosis, competition, induced systemic resistance, and siderophore production [29,30,31]. Several mechanisms used by PGPR in plant growth promotion are described in detail in the following section. 

## 3. Mechanisms of Action of PGPR in Plant Growth Promotion

### 3.1. Biological Nitrogen Fixation

Nitrogen, one of the most vital elements, plays an important role in plant growth and various metabolic activities. Nitrogen (N_2_) accounts for 78% of total atmospheric gasses, but this form is not available to plants. Conversion of nitrogen to ammonia (NH_3_) by certain bacteria and archaea using a nitrogenase protein complex [32] is termed biological nitrogen fixation (BNF) [7,33]. Various nitrogen fixers are distributed in nature, and the N_2_ fixing ability of microbes can be a substitute for commercial fertilizers [34,35]. Nitrogen-fixing microbes can be classified as (i) symbiotic (mutualistic relationship between bacteria and leguminous plants and non-leguminous trees) and (ii) non-symbiotic (free-living and endophytic organisms) [7,36,37,38]. Widely reported symbiotic N_2_ fixers in soil are *Frankia* and *Rhizobium* species, whereas diazotrophic PGPR, including *Cyanobacteria*, *Azospirillum*, *Pseudomonas*, *Azotobacter*, *Acetobacter*, and *Nostoc*, are non-symbiotic nitrogen-fixing microbes [18,36]. Non-symbiotic microbes are important in the natural environment; they fix less nitrogen but provide enough to meet host plant demand. A complex process of infection and establishment between the roots of leguminous plants and symbionts results in the formation of root nodules [39]. Energy in the form of ATP (adenosine triphosphate) is required in the process of nitrogen fixation: for this, bacterial carbon resources undergo oxidative phosphorylation, and the energy is stored in the form of glycogen [40]. The genes for nitrogen fixation in both symbionts and free-living non-symbionts are *nif* (nitrogenase complex) genes [41]. The *nif* genes encode enzymes involved in the fixation of atmospheric N_2_ into a form of nitrogen available to plants. The primary enzyme encoded by *nif* genes is the nitrogenase complex. In addition to enzymes, *nif* genes also encode regulatory proteins for N_2_ fixation. The oxygen (O_2_) concentration and low level of nitrogen in the root environment of the host plant are responsible for the induction of *nif* gene expression [40]. Oxygen is a negative regulator of *nif* genes and has an inhibitory effect on nitrogenase enzyme activity, though it is required for the respiration of *Rhizobium* and bacteroid species. Bacterial hemoglobin, which binds free oxygen radicals, provides sufficient O_2_ for bacteroid respiration within root nodules while simultaneously preventing O_2_ from inhibiting N_2_ fixation [36]. At a low level of dissolved oxygen, after the transformation of *Rhizobium etli* with a hemoglobin gene from *Vitreoscilla* sp. (a Gram –ve bacterium), the respiratory rate of rhizobial cells was increased 2–3-fold compared with non-transformed strains [38]. 

The nitrogenase complex (*nif*) consists of regulatory genes, structural genes, and genes involved in the biosynthesis of Fe protein and Fe–molybdenum cofactor activation. In non-symbiotic microbes, *nif* genes are found in a cluster of around 20–24 kb containing seven operons encoding 20 proteins. [42]. A small rise in the ethylene level of a plant may cause infection and nodulation by *Rhizobium* species in the roots of leguminous crops [7]. However, the increase in ethylene levels reduces nodulation in legumes [43]. Some rhizobacterium strains have the ability to mitigate the rise in ethylene levels by synthesizing rhizobiotoxine (a small molecule) in the host legume roots [7,14], which inhibits the function of the ACC synthase enzyme (ethylene biosynthetic enzyme). Some ACC deaminase-synthesizing rhizobacteria remove ACC, which is the precursor of ethylene in green plants [43]. The nodulation efficacy of *Rhizobium* spp. in which ACC deaminase is absent can be increased by inserting isolated genes of ACC deaminase. For instance, a *nif* gene from *R. leguminosarum* bv. *viciae* was isolated and inserted into the DNA of *Sinorhizobium meliloti*, and it dramatically increased the biomass and nodulation of alfalfa plants [44]. A summary of a range of studies regarding the PGPR-mediated growth enhancement of various crops through BNF is reported in Table 1.

### 3.2. Phosphorus Solubilization

Phosphorus (P) is the second most vital macroelement after nitrogen and is required for adequate plant nutrition [18]. It has key roles in the processes of photosynthesis, energy transfer, and various plant metabolic processes [67]. In most soils, P is present in excess, but not in a form available to plants [68]: only 0.1% of total soil phosphorus is available for plant use [69,70,71]. Phosphorus becomes immobilized in soil through complex interactions with various cations such as Ca^2+^ and Mg^2+^ in alkali soils and Fe^3+^ and Al^+^ in acidic soils [7,72]. In most soils, available P is not present in a sufficient quantity for plants, and commercial fertilizers are used to meet plant requirements [73]. According to estimates, about 52.3 billion tons of phosphorus fertilizers are applied to agricultural land every year [74]. Excessive use of synthetic fertilizers not only results in environmental hazards, i.e., global warming due to the production of nitrogen oxides, but is also a source of eutrophication of water bodies [75,76]. Plant growth-promoting microbes, on the other hand, offer a suitable option to enhance crop productivity with no adverse effects on the environment. 

These microbes increase phosphorus availability in soil by mediating the mineralization of organic P in soil [77]. Mineralization of organic P by microorganisms requires P solubilization, which greatly depends on soil pH. Such microbes modify the soil pH by producing various organic and inorganic acids and other metabolites through a mechanism known as rhizosphere acidification. Certain *Rhizobium*, *Pseudomonas*, and *Bacillus* species are considered phosphate-solubilizing bacteria (PSB) [18]. However, the exact extent of P solubilization is highly variable among soils and the various bacteria involved and further depends upon the soil conditions. Recently, Muleta et al. [78] reported that various strains of *Mesorhizobium* sp. caused the pH to drop from 6.9 to 5.2 in Pikovskaya’s medium amended with tri-calcium phosphate, and, as a result, the P solubilization increased as desired, from 1.53 to 138 µg mL^−1^. In the current situation, it is necessary to explore an economically affordable and ecologically healthy option for P availability in roots and plants. Keeping this scenario in mind, there should be an emphasis on the use of PGPR instead of abundant reliance on the use of commercial phosphorus sources. 

Various microbial species, including bacteria and fungi, are involved in P solubilization in soil [69,79]. The integration of organic amendments with a low dose of chemical fertilizer is a strategy to reduce the soil C:P ratio and increase P availability for crop plants [80]. About 50% of all bacteria are able to solubilize P [68]. *Bacillus*, *Rhizobium*, *Pseudomonas*, *Azotobacter*, *Burkholderia*, *Enterobacter*, *Microbacterium*, *Serratia*, *Burkholderia*, and *Beijerinckia* are the most significant PSB [81]. Some soil bacteria, e.g., actinobacteria, release low-molecular-weight organic solutes and solubilize inorganic phosphate [36]. The principal P solubilization mechanism involves the production of organic acids and OH^−^ ions, production of protons or bicarbonate release (cation/anion balance), gaseous exchange of O_2_/CO_2_, and production of siderophores by soil microbes [82]. The release of organic acid by microorganisms to solubilize inorganic P bound to soil colloids is an important mechanism in which COO^−^ (carboxyl group) and OH^−^ (hydroxyl ion) act as chelators of cations such as Fe, Al^3+^, and Ca^2+^ and compete for P adsorption sites in soil [67,75,83,84]. Chelators are low-molecular-weight organic acids that compete with Al and Fe oxides for P fixation sites [85]. Gluconic and ketogluconic acids are known to act as Ca chelators in alkaline soils [86]. Organic acids are released by microbial biomass during fermentation or oxidative respiration of organic sources [82]. Phosphate-solubilizing microbes primarily secrete oxalic, malic, fumaric, acetic, tartaric, malonic, glutanic, propoinic, butyric, lactic, gluconic, 2-keto gluconic, glyconic, and oxalic acids [82,87]. These low-molecular-weight organic acids solubilize the fixed inorganic P by lowering the soil pH, chelating cations, and competing with phosphate (PO_4_^−^) for adsorption sites in the soil [88]. Excretion of root exudates, e.g., ligands, also plays a significant role in altering P concentration in soil solution [89]. Inorganic acids (e.g., hydrochloric acid) can also solubilize PO_4_^−^ but are less effective than organic acids at the same pH [90]. Hence, phosphorus solubilization is an effective and sustainable approach to enhance the P availability for plants and reduce dependence on unsustainable, costly synthetic fertilizer sources. The integrated use of organic sources together with reduced doses of inorganic fertilizer increases the removal of P fixed with soil colloids and sustains soil fertility [91]. For sustainable soil health and fertility, research is required to enhance the efficiency of P solubilization approaches and identify new bacteria species with more efficiency. A summary of various studies involving P solubilization through microbial candidates and improvement in plant growth is provided in Table 1.

### 3.3. Potassium Solubilization

Another vital macronutrient after nitrogen and phosphorus is potassium (K), which is the seventh most abundant element in the Earth’s crust. It plays a key role in various metabolic, growth, and development-related processes in plants. Moreover, potassium is responsible for activating more than 80 enzymes involved in photosynthesis activity, nitrate reduction, starch synthesis, and various energy metabolic processes [92,93,94]. An increase in plant vigor against various biotic stresses, diseases, and pests is also regulated by an adequate supply of K [95]. The potassium concentration in soil ranges between 0.04% and 3%, but only 1–2% of the total concentration is available to plants, and 98–99% is fixed in its mineral form [96]. The remaining 90–98% of soil K is fixed with minerals and termed mineral K, which is unavailable to plants. In soil, K is found in various forms, including solution K, mineral K, exchangeable K, and non-exchangeable K [96]. The solution form of K is promptly consumed by plants and microbes and might leach down in some soils. Generally, in agricultural soils, solution K ranges from 2 to 5 mg L^−1^. Minerals containing K are feldspar and mica. Unexchangeable K reacts with oxygen atoms in the interlayers of certain clay minerals and becomes fixed. This form of K comprises 1–10% of total soil potassium [97]. 

Nutrient acquisition in soil is enhanced by microbial activity via the processes of decomposition, mineralization, and storage [52]. Integrated use of synthetic and organic sources in various moisture regimes is important to sustain soil physical health, nutrient availability, microbial activity, and crop production [91]. Potassium-solubilizing microbes are considered major contributors to K dissolution in soil, and researchers have also used strains of potassium-solubilizing bacteria to improve nutrient availability and crop yield [98]. Saprophytic bacteria, actinomycetes, and fungi are known as K-solubilizing microbes [99]. Potassium-solubilizing bacteria are found in the root rhizosphere [100]. Potassium-solubilizing bacterial isolates with more efficiency still need to be screened with the use of advanced Aleksandrov medium, which largely depends on the halo zone structure adjoining the bacterial colonies [101,102]. The solubilization of rock silicate by various bacterial species is well known. In soil bacterial groups, *Bacillus mucilaginosus*, *B. edaphicus*, and *B. circulans* are the most efficient K solubilizers. Potassium solubilization by microbes is affected by pH, aeration, the bacterial strains utilized, and the type of K-bearing natural resources. Hence, a slight increase in soil pH dramatically changes the solubilization of silicate [103]. 

Low-molecular-weight organic acids, including tartaric acid, lactic acid, fumaric acid, glycolic acid, oxalic acid, malic acid, citric acid, gluconic acid, and 2-ketogluconic acid, are produced by KSB and are able to release fixed K from K-bearing minerals [58,99,104]. Gasses such as ammonia (NH_3_) and H_2_S (hydrogen sulfide) are released as a result of organic matter decomposition which, after oxidation, form strong acids such as H_2_SO_4_ (sulfuric acid) and HNO_3_ nitric acid. Cations such as K^+^, Ca^2+^, Mn^2+^, and Mg^2+^ are displaced from the cation exchange site by H+ ions [105]. KSB produce organic acids that help to release K from mineral complexes by forming chelates with Fe^2+^, Al^3+^, Si^4+^, and Ca^2+^ ions bound to K minerals [106]. According to one study [107], weathering of phlogopite is facilitated by KSB through acidic dissolution of the crystal lattice and Al^3+^ (aluminum) chelation. Moreover, isolated strains of *B. altitudinis* can hasten the weathering of K-containing feldspar mineral, change surface morphology, and induce the development of a novel mineral complex. One strain dissolved K in the mineral (feldspar) by producing organic acids and released considerably more Al, Fe, and Si [105]. It has been observed that the assembly of extracellular polymers, such as proteins and polysaccharides, affects mineral dissolution and leads to production of the available form of K from K-bearing soil minerals that can be taken up by plants [108,109]. KSB create a microenvironment by synthesizing biofilms around the microbial cells for the purpose of weathering [106]. Due to biofilm formation, the residence time of water on aluminosilicate will be high compared with the residence time on bare rock, ultimately leading to high mineral weathering. Microbial biofilms hasten the process of weathering in addition to decreasing nutrient losses from minerals by acting as a defensive layer on the mineral. Furthermore, the formation of a biofilm on the mineral surface promotes the decay of K-rich shale and the release of K, Al, and Si in the bacteria–mineral contact model [110]. The use of compost not only increases the activity of potassium-solubilizing microbes but also acts as a source of mineral potassium that is released into soil [111]. According to Wang et al. [112], plant roots release organic acid, which is effective in mobilizing bound K. Other PGPR (e.g., IAA-producing bacteria) may also play a role by providing K to plants through an increase in root exudate formation [113]. A summary of K-solubilizing microbes involved in plant growth promotion is given in Table 1.

### 3.4. Siderophore Production

Siderophores are small organic molecules formed by microorganisms in iron-deficient conditions that improve the ability to uptake iron [114]. As a micronutrient, iron is important for the survival of all organisms. The predominant form of iron present on Earth is Fe^3+^, but this form is poorly soluble; therefore, the amount of Fe available to living organisms is very small. Microorganisms and plants need a sufficient amount of Fe, which is of greater concern in the root region, where plants and microbes compete for Fe [115]. PGPR generate siderophores of low molecular weight in an iron-limiting environment in order to make iron available to plants [116]. Thus, they increase plant growth by enhancing iron availability in the rhizospheric zone [117]. The siderophores create Fe competition in the rhizospheric zone, which decreases pathogenic microbe abundance [114,118] and increases plant growth. Siderophores produced by PGPR improve plant Fe uptake and reduce the growth of pathogens by showing a high affinity for rhizospheric Fe^3+^ and retaining almost all free iron [119]. 

Siderophores can be divided into four main groups based on their functional groups: hydroxamates, catecholates, carboxylates, and salicylates [120]. The beneficial effects of siderophores produced by PGPR on plant growth have been demonstrated in many studies (Table 1). For example, in some studies, radiolabeled ferric siderophores were used as a source of iron, and plants were able to uptake the labeled iron [116]. When mung bean plants were inoculated with siderophore-producing *Pseudomonas* strain GRP3 and grown in iron-deficient soils, chlorotic symptoms were reduced, and the chlorophyll level increased as compared to the control plants [121]. In another study, *P. fluorescens* C7 Fe-pyoverdine was taken up by *Arabidopsis thaliana* plants, resulting in increased iron content in plant tissues [78]. In ecologically stressed conditions, such as pollution with heavy metals, the iron supply is very important. In such situations, siderophores alleviate the harmful effects of heavy metals on plants [122]. 

### 3.5. Production of Phytohormones

Phytohormones or plant growth regulators are organic constituents that promote plant growth [123]. PGPR stimulate the production of phytohormones in plants and hence promote their growth and development. Various bacteria performing this role have been isolated and characterized (Table 1), and promising results in crop growth promotion have been reported. Phytohormones such as gibberellins, cytokinin, abscisic acid, ethylene, and auxin can promote the blooming of root cells via the overproduction of adjacent roots, with a subsequent increase in nutrient and water uptake [124]. 

The system that plants use to endure stress is complex and intricate. Microorganisms use different biochemical and molecular mechanisms to improve plant growth. PGPR improve plant growth by adjusting hormones and the accessibility of nutrients in plants and stimulating resistance to disease-causing organisms [15,125]. These also generate specific metabolites that control plant pathogens in the root zone. For example, rhizobitoxine increases plant growth and expansion in stressed conditions by limiting ethylene production [126]. In addition, different bacteria have sigma factors that modify the expression of plant genes in a way that increases their survival in stress situations [26]. 

The most commonly studied phytohormone produced by PGPR is indole-3-acetic acid (IAA), which facilitates plant–microbe association [127,128]. The role of IAA depends on the endogenous IAA levels in plants [111]. Auxins produced by PGPR are involved in plant growth promotion by stimulating transcriptional alterations in hormones [129], enhancing root biomass, shrinking stomata size and density [130], and triggering the expression of auxin response genes [131].

Cytokinins and gibberellins are also produced by various PGPR (Table 1) [26,132]. Several strains of PGPR can stimulate a comparatively huge volume of gibberellins, leading to increased plant shoot growth [133]. The association of these hormones with auxins can modify the root structure [134]. PGPR also produce cytokinins, which increase the production of root exudates by the plant [131], perhaps increasing the presence of PGPR that are associated with the plant. 

The role of PGPR in stressed and unstressed environments has been studied and they frequently appear to provide better growth stimulation in stressful situations, such as drought stress [135]. For various PGPR, ethylene plays a key role in increasing plant tolerance under stress conditions [136]. Several studies have shown improved stress tolerance in plants after inoculation with PGPR that produce 1-aminoacyclopropane-1-carboxylase (ACC) deaminase [135,137]. This seems to arise because PGPR are able to prevent ethylene from reaching levels that decrease plant growth [131,138], which has been tested with *Camelina sativa* [139]. 

Furthermore, working as biocontrol agents, PGPR protect plants against pathogens by producing biochemical and molecular defense responses in the plant [140]. PGPR can activate ISR within plants, which stimulates the expression pathogenesis-related genes, facilitated via phytohormone signaling pathways and defense regulatory proteins, to protect plants against future pathogen attacks [141]. 

### 3.6. Root Colonization and Increased Uptake of Plant Nutrients

Plant roots interact with a wide range of soil microorganisms inhabiting the rhizosphere [9,142]. An important aspect of such associations between the plant roots, rhizosphere, and rhizobacteria is the improvement in root growth and proliferation, which play significant roles in the transfer of nutrients and water to the upper plant parts [9,143,144]. For instance, such an association results in enhanced root exudation which, in turn, attracts microbial candidates toward the vicinity of the root, which eventually results in aggressive root colonization [145,146,147].

The concept of root colonization denotes the multiplication of bacterial populations ectophytically in the rhizosphere and endophytically inside the roots [148]. Root colonization by PGPR is considered essential for plant development. In the process of root colonization, rhizobacteria propagate from the source of the inoculum, such as in seed treatments, to the actively growing root region and proliferate in the rhizosphere [149]. Roots play an important role in the uptake of essential nutrients needed for plant growth and survival. In the rhizosphere, aggressive root colonization enhances plant growth, which is indicative of positive root–rhizosphere–rhizobacterial interaction [119]. Moreover, several rhizobacteria produce one or more types of phytohormones in the rhizosphere (Table 1) that also activate phytohormone-producing genes through organic compounds abundant in the root cap and the elongation region [145,148]. For example, Lakshmanan et al. [150] observed that when *B. subtilis* strain FB17 was inoculated in the roots of *Arabidopsis thaliana*, it expressed multiple genes involved in metabolism, stress response, and plant defense during the root colonization process. 

## 4. Plant Growth-Promoting Rhizobacteria (PGPR) as Biocontrol Agents: An Overview

In order of decreasing abundance, bacteria, actinomycetes, fungi, protozoa, nematodes, and microarthropods are among the microbes found in the rhizosphere. The plant rhizosphere is a thin layer of soil that adheres to the root surface [132,151]. Plant-associated microbes impact the growth, yields, and physiological and environmental benefits of the host plant in a variety of ways. 

Plant disease outbreaks are a major cause of decreased crop yield, deteriorating production quality, and causing the contamination of food grains. Pesticides have been developed in response to the ever-increasing range and complexity of plant diseases [152,153]. Unfortunately, continued use of these pesticides has resulted in phytopathogen resistance, which raises a number of environmental concerns. Biological control is being considered as an alternative to pesticides for phytopathogen control. [154]. Plant growth and health are assisted by the use of PGPB as biological agents. PGPB have a number of benefits over traditional pest control methods. The use of PGPB in agriculture is both environmentally friendly and non-toxic. PGPB work through a variety of mechanisms to reduce or avoid harm caused by phytopathogens [24]. 

Plant growth is influenced by PGPR in two ways: indirectly and directly. Direct plant growth promotion by PGPR involves either providing the plant with bacterium-produced compounds, such as phytohormones, or promoting the absorption of certain nutrients from the environment [41]. As PGPR reduce or prevent the negative effects of one or more phytopathogenic species, they indirectly promote plant growth. This can be accomplished by generating antagonistic compounds or inducing pathogen resistance [41]. One or more of these mechanisms can be used by a specific PGPR to influence plant growth and development.

PGPR may function as biocontrol agents via a variety of mechanisms (Table 2) irrespective of their position in plant growth enhancement, such as the establishment of auxin phytohormone development [51], reduction in plant ethylene levels [155], or nitrogen fixation [156]. Plant–PGPR interactions are commercially exploited [157], and they hold great promise for long-term agriculture. A number of commercial and food crops have been studied in relation to these associations [158]. 

Furthermore, siderophore synthesis is one of the most important PGPR mechanisms for preventing phytopathogen propagation. These siderophores serve as iron chelators, binding the majority of the iron in the rhizosphere. As a result, the lack of iron in the rhizosphere inhibits bacterial and fungal pathogen proliferation [24]. Systemic resistance to phytopathogens is another potential mechanism used by PGPR in pathogen biocontrol.

The use of bacteriophage as a biocontrol agent has been a promising yet uncommon technique in recent years. Phages have the inherent ability to address phage resistance or new bacterial strains and are compatible with a variety of other biocontrol agents. Because of their sensitivity to UV light, they must be sprayed on the plant in the evening [166]. They can be used in phage-based diagnostics of phytopathogenic bacteria in addition to being used as biocontrol agents. 

Because of the negative effects of chemical pesticides on both ecosystems and humans, sustainable agriculture has become a global necessity in recent years [10]. Various studies have investigated the use of PGPR in increasing crop production, revealing their potential to improve crop nutrition, yield, and disease management [12,132,167,168]. The use of PGPR has allowed decreasing chemical inputs into the soil and helped to mitigate environmental hazards caused by the overuse of chemicals [169,170]. Singh et al. [167,168,171] have identified PGPR as one of the suitable alternatives for use as growth promoters and biocontrol agents.

## 5. Mechanisms of Action of PGPR as Biocontrol Agents

### 5.1. Production of Antibiotics

The key method of plant growth-promoting bacteria for combating phytopathogen-caused damage is the production of antibiotics (Figure 1, Table 2). The biocontrol abilities of *Pseudomonas* strains are largely dependent on aggressive root colonization, induction of plant systemic resistance, and the development of antifungal antibiotics [172]. The potential of rhizobacteria as biocontrol candidates against phytopathogens is generally associated with the development of one or more antibiotics [155]. Over the last two decades, the concept of antibiosis, or biocontrol based on the synthesis of molecules that destroy or slow the growth of the target pathogen, has become well defined [140,173,174]. Antibiotics are a diverse group of organic, low-molecular-weight compounds that inhibit the growth or metabolic activities of bacteria and other microorganisms [175]. According to [176], six groups of antibiotic compounds are best linked to the biocontrol of root diseases: phenazines, phloroglucinols, pyoluteorin, pyrrolnitrin, cyclic lipopeptides, and hydrogen cyanide (HCN), all of which are diffusible except for HCN, which is volatile. Lipopeptide biosurfactants developed by *Pseudomonas* and *Bacillus* species have recently been applied in biocontrol because of their possible beneficial impact on competitive interactions with bacteria, fungi, oomycetes, protozoa, nematodes, and plants [177,178]. Antibiotics produced by *Pseudomonads* include 2,4-diacetylphloroglucinol (DAPG), amphisin, hydrogen cyanide, phenazine, oomycin A, tropolone, pyoluteorin, tensin, pyrrolnitrin, and cyclic lipopeptides, while *Streptomyces, Bacillus*, and *Stenotrophomonas* spp. produce kanosamine, oligomycin A, xanthobaccin, and zwittermicin, which have been identified as antibiotics that have antibacterial, antifungal, antiviral, anthelminthic, antimicrobial, cytotoxic, phytotoxic, antioxidant, and antitumor properties [179].

Antibiotics have been isolated from a wide range of fungal and bacterial species, with mechanisms of action that include inhibiting pathogen cell wall synthesis, influencing cell membrane structures, and inhibiting the development of initiation complexes on the small subunit of the ribosome (Table 2) [180]. Pyrrolnitrin, an antibiotic developed by the *P. fluorescens* BL915 strain, has been shown to protect cotton plants from *Rhizoctonia solani* during damping-off [181].

Antibiotics produced by the majority of *Bacillus* species, such as polymyxin, circulin, and colistin, are effective against Gram-positive and Gram-negative bacteria as well as many pathogenic fungi [180]. The *B. cereus* UW85 strain contributes to the biocontrol of damping off in alfalfa by suppressing oomycete pathogens and producing the antibiotics zwittermicin A (aminopolyol) and kanosamine (aminoglycoside) [182,183]. Some researchers have highlighted the use of sporulating Gram-positive species, such as *Bacillus* and *Paenibacillus* spp., as biocontrol agents to serve as a biological solution, which can confer higher population stability during the formulation and storage of inoculant products [184,185].

### 5.2. Induction of Plant Systemic Resistance

Non-pathogenic rhizobacteria are said to suppress disease in plants by inducing a resistance mechanism known as induced systemic resistance (ISR) [186]. When plants are properly stimulated, they gain an enhanced defensive capacity, which is known as induced resistance [186]. Van Peer et al. [187] identified ISR in carnation plants that were systemically protected against *F. oxysporum* by the *P. fluorescens* strain WCS417r, and in [188], the leaves of cucumber plants with rhizobacterial strains were protected against anthracnose caused by *Colletotrichum orbiculare*. The pathogens and the studied rhizobacteria were inoculated and remained confined and spatially isolated on the same plant, preventing microbial antagonism and allowing the protective effect to be mediated by the plant.

The ISR is a central mechanism for *Pseudomonas*, *Trichoderma*, *Bacillus*, and mycorrhiza to improve plant protection against various pathogens. ISR is the product of pathogen-specific recognition by plant receptors [141,179]. Salicylic acid is generated by several PGPB, which activate systemic acquired resistance (SAR), a mechanism similar to ISR. Another important biocontrol mechanism is phytopathogen virulence factor detoxification. Toxins formed by *Xanthomonas albilineans* and *Fusarium* species can be detoxified by certain PGPR [154]. Quorum sensing is used by many PGPR to control virulence factor development, which inhibit the pathogen’s quorum sensing ability by interfering with autoinducer signals, halting the expression of virulence factors [24].

Rhizobacteria-mediated ISR is similar to pathogen-induced systemic acquired resistance (SAR) in that both types of induced resistance result in uninfected plant parts becoming more resistant to plant pathogens, such as fungal, bacterial, and viral pathogens as well as nematodes and insects [186,189,190,191]. In the same plant, the same strain causes resistance to multiple pathogens [192]. *Pseudomonas* and *Bacillus* spp. are the most studied rhizobacteria that cause ISR [193,194,195]. Vleesschauwer and Höfte [192] coined the term ISR to describe induced systemic resistance caused by non-pathogenic rhizobacteria or PGPR, regardless of the signaling mechanism involved, while SAR refers to salicylic acid-dependent induced resistance caused by a localized infection.

In several plant species, the ability to develop ISR in response to specific rhizobacteria has been demonstrated [186] and appears to be dependent on the specificity of the rhizobacteria–plant interaction [196]. Failure to induce ISR in certain hosts may be due to a lack of inducing components being generated in the rhizosphere or a plant species’ inability to perceive certain compounds [196]. The evidence suggests that induction of resistance requires specific identification between the plant and the rhizobacteria. *Pseudomonas* putida WCS358r and *Pseudomonas fluorescens* WCS374r, for example, behave differently depending on the plant species: WCS358r elicits ISR in *Arabidopsis*, but not in radish or carnation plants [187,194,197,198]. Radish plants, on the other hand, react to WCS374r, while *Arabidopsis* does not [194,198]. 

### 5.3. Rhizosphere Competence and Root Colonization

For bacteria to be considered valid PGPR, root colonization is needed, and it is widely assumed that a biocontrol agent must colonize the rhizosphere and the surface of the plant that it protects [199,200,201]. As a result, if a PGPR species does not effectively colonize the roots, it is also ineffective as a biocontrol agent against root disease [202].

The most common root-colonizing PGPR in various crops are *Pseudomonas* and *Bacillus* spp. (Table 2). Several members of this group have widespread soil distribution, are effective rhizosphere colonizers, and produce a variety of metabolites that inhibit a wide range of pathogens in plants [203]. Many other root-colonizing PGPR strains have also been discovered to have antifungal properties against a variety of soil pathogens.

Biological regulation of soilborne diseases is also inconsistent among several possible biocontrol strains, such as *Pseudomonas* and *Bacillus* spp. Inadequate root colonization by introduced bacteria is one of the major causes of this inconsistency [204]. Mutants of *Pseudomonas* strains that had lost their biocontrol activity serve in verifying the connection between the poor biocontrol output of a biocontrol agent and inefficient root colonization. Understanding the bacterial traits that lead to root colonization is critical in this regard.

Molecular techniques can now be used to identify and count microorganisms on plant surfaces in real time. One of the methods used in the study of in situ bacterial root colonization was the use of marker genes, such as the *gfp* gene, which encodes the green fluorescent protein (GFP). Confocal laser scanning microscopy (CLSM) can be used to monitor and visualize GFP-transformed bacteria [204]. This GFP-based technique can be used to research the colonization patterns of various biocontrol agents in addition to visualizing root colonization.

### 5.4. Outcompetition and Direct Antagonism Against Pathogens

Biocontrol agents are bacteria that minimize the occurrence or severity of plant diseases, while antagonists are bacteria that have antagonistic behavior against a pathogen [205]. The following bacterial antagonistic behaviors and rhizospheric climates can be highlighted: (1) synthesis of hydrolytic enzymes that can lyse pathogenic fungal cells, such as chitinases, glucanases, proteases, and lipases [180,206], (2) competition for nutrients and appropriate colonization of niches at the root surface [207,208,209], (3) control of plant ethylene levels through the ACC deaminase enzyme, which can act to modulate ethylene levels in a plant in response to infection-induced stress [186,210], and (4) development of siderophores and antibiotics. The development of secondary metabolites, such as auxins, IAA, cytokinins, riboflavin, and vitamins, may be a direct result of the PGPR stimulation of plant growth [211]. Cell division and expansion [212] or improved nutrient availability [41,213,214,215] stimulate plant organ development.

### 5.5. Synthesis of Hydrogen Cyanide (HCN)

Biocontrol mechanisms of bacteria, such as those present in some *Pseudomonas* strains, are typically dependent on secreted bioactive factors that target the pathogen, such as antibiotics, exoenzymes, or HCN, according to Thomashow and Weller [216]. Dekkers et al. [217] discovered that phenazine-1-carboxamide (oxychlororaphin, or OCP), a phenazine formed by *P. chlororaphis* PCL1391, prevented *F. oxysporum* f.sp. *radicis-lycopersici* from causing tomato root rot. Because of their rapid and violent colonization of plant roots, fluorescent pseudomonads have been considered effective biological control agents against soilborne plant pathogens, according to Lugtenberg et al. [218]. They observed that one process was competition for nutrients in the rhizosphere at favored colonization sites, while another was the development of metabolites, including antibiotics, siderophores, and hydrogen cyanide. According to Kremer and Souissi [219], rhizobacteria strains can synthesize hydrogen cyanide and have an impact on seedling root growth in a variety of plants. They discovered that about 32% of bacteria in a sample of over 2000 isolates were cyanogenic, with HCN levels ranging from trace to >30 nmol/mg cellular protein. *Pseudomonads* were found to be the most susceptible to cyanogenesis, which was aided by the addition of glycine to the culture medium.

### 5.6. Synthesis of Cell Wall Degrading Enzymes

The development of cell wall degrading enzymes is one of the main mechanisms used by biocontrol agents to regulate soilborne pathogens [220,221]. Cell wall degrading enzymes secreted by biocontrol strains of PGPR, such as β-1,3-glucanase, chitinase, cellulase, and protease, have a strong inhibitory impact on the hyphal growth of fungal pathogens. Chitinase and β-1,3-glucanase are enzymes that break down chitin, a soluble linear polymer of β-,4-N-acetylglucosamine, which is a major component of fungal cell walls.

*Paenibacillus* and *Streptomyces* spp. produce β-1,3-glucanase, which lyses the fungal cell walls of pathogenic *F. oxysporum*. *Bacillus cepacia* produces b-1,3-glucanase, which breaks down the cell walls of soilborne pathogens such as *R. solani*, *P. ultimum*, and *S. rolfsii* [48]. *B. licheniformis*, *B. cereus*, *B. circulans*, and *B. thuringiensis* are all potential biocontrol agents with chitinolytic activity [222]. *Serratia marcescens*, *Enterobacter agglomerans*, *Pseudomonas aeruginosa*, and *Pseudomonas fluorescens* have been found to have chitinolytic activities among Gram-negative bacteria [223]. 

The structural integrity of the target pathogen’s cell walls is affected by rhizobacteria cell wall degrading enzymes [224]. The chitinolytic and antifungal activities of *S. marcescens* B2, a potent biocontrol strain, against the soilborne pathogens *R. solani* and *F. oxysporum* were investigated by Someya et al. [225]. The mycelia of fungal pathogens co-inoculated with this strain displayed a variety of abnormalities, including partial swelling in the hyphae and at the tip, hyphal curling, and hyphal tip bursting.

Several bacteria produce enzymes that hydrolyze cellulose, hemicelluloses, chitin, and proteins, which inhibit phytopathogen activity. *Serratia plymuthica* C48, *Serratia marcescens*, *Paenibacillus* sp., *Streptomyces* sp., and *Pseudomonas stutzeri* produce chitinase, which degrades the mycelia of different fungal phytopathogens. *Streptomyces*, *Paenibacillus*, and *Bacillus* sp. produce β-1,3-glucanase, which lyses fungal cell walls. PGPR protease and lipase can also degrade proteins and lipids associated with cell walls. Hydrogen cyanide is produced by *Pseudomonas*, *Rhizobium*, *Bacillus, Alcaligenes*, and *Aeromonas* and improves the antifungal activity of these bacteria [12,24,48,152,179].

## 6. Plant Growth-Promoting Rhizobacteria (PGPR) in Bioremediation: An Overview

**Figure 2:**
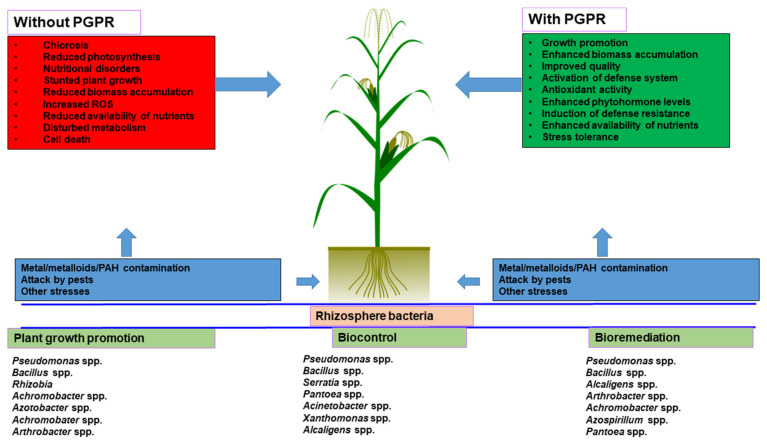
Consequences of stresses on plants without PGPR (left side) and with PGPR (right side). The bottom part shows the most studied microbial candidates involved in plant growth promotion, biocontrol, and bioremediation processes.

High concentrations of metalloids and trace metals can contaminate soil as a result of pollution from the rapidly developing industrial sector, dumping of petrochemical spillage, trace metal waste, leaded fuel, mine tailings, pesticides, sewage sludge, atmospheric deposition, and coal combustion residues [226,227]. Arsenic, chromium, cadmium, lead, nickel, copper, mercury, and zinc are the most common inorganic pollutants found in industrial wastes [16]. Organic contaminants (i.e., antibiotics such as tetracyclines, sulfonamides, macrolides, and quinolones), pesticides (i.e., bentazon and atrazine), polycyclic aromatic hydrocarbons (PAHs), total petroleum hydrocarbons (TPHs) from the consumption and exploration of fossil fuels, polychlorinated biphenyls (PCBs), halogenated compounds (i.e., trichloroethylene and perchloroethylene), and polychlorinated terphenyls (PCTs) are extensively used in industrial sectors and are considered most resistant [228,229,230,231]. Pesticides also introduce a number of molecular, morphological, physiological, and biochemical changes in plants that adversely affect productivity and growth in addition to the development of pest resistance [232]. Thus, in order to clean up trace metals and polluted environments, bioremediation and remediation technologies are constantly being improved, using naturally occurring or genetically engineered microorganisms [233,234]. According to recent research, among microorganisms used for bioremediation, the utilization of plant growth-promoting rhizobacteria (PGPR) is becoming more common due to their various abilities to detoxify and degrade toxins, as well as their significant effects on plant growth promotion [229,231]. In contrast to physical and chemical remediation approaches, bioremediation with PGPR is gaining more attention for the removal of industrial waste contaminants due to its environmentally friendly nature, lower cost, and demonstrated performance [230]. The ability of PGPR to improve plant growth and overcome trace metal toxicity can be aided by their interactions [227]. Microbes such as PGPR colonize the rhizosphere or live near the surface of roots, and they tend to produce and secrete a variety of regulatory compounds, i.e., metal-binding proteins, phytohormones, and siderophores, to protect the plant from toxicity [233]. Similarly, dark brown sugarcane (*Saccharum officinarum* L.) molasses, because it is rich in complex organic compounds and trace metals, inhibits seed germination and depletes vegetation on agricultural soils by lowering the alkalinity of soil and the availability of manganese [235,236]. Recently, PGPR were used in the bioremediation of sugarcane molasses-based anaerobically digested distillery effluent [237]. Distillery spent wash disposed on land is considered very hazardous waste, causing a decrease in the alkalinity of soil, seed germination inhibition, and vegetative destruction [231]. Moreover, it also lowers the penetration of sunlight in marine environments, thereby lowering the photosynthetic activity and dissolved oxygen content and harming both aquatic flora and fauna [233,235]. Correspondingly, the ability of grasses and native weeds to remove trace metals by using in situ phytoremediation of distillery waste was also observed [238]. Previously, a number of rhizosphere bacteria were screened and tested for their bioremediation potential against inorganic and organic pollutants from the soil, as shown in (Table 3 and Table 4). Bacterial pretreatment of agricultural effluents mediates the modification and oxidation of inorganic and organic contaminants, making them readily bioavailable to wetland plant roots and rhizosphere microorganisms, the latter of which use the biotransformed materials as biomass, nitrogen, and energy supply [239,240,241]. Rhizosphere bacteria are equipped with many mechanisms, including bioaccumulation (net accumulation of contaminants in microorganism cells), biomineralization (transfer of aqueous contaminants into crystalline or amorphous precipitates), biotransformation (transformation of contaminants from toxic to less toxic forms), and biosorption (binding of contaminants with cation binding proteins present on the cell wall of microorganisms) [3,7,16,231]. To overcome the toxicity of trace metals in the rhizosphere, microorganisms have developed a range of pathways, including (1) transporting them to the exterior of the cell by metal ion pumping [242], (2) sequestration and accumulation of metal ions inside the cell [243], (3) transformation/conversion of toxic metals to a less toxic form [244], and (4) desorption/adsorption of metals [245]. Root exudates play a significant role in bacterial quorum sensing and biofilm formation because they can chemotactically attract rhizobia to plants, resulting in colonization and adherence to the legume roots along with the control of genes involved in rhizobial nodulations (rhizosphere and nod-expressed (rhi)), which enhances the development of plants and bacterial remediation potential [241,246]. Overall, trace metals are mobilized during phytodegradation via acidification, chelation, and protonation, while they are immobilized via alkalinization, complexation, and precipitation [234,237,247]. Through a variety of plant growth pathways, PGPR may be used to maximize crop yields [245]. Environmentally sustainable methods have encouraged the utilization of a wide variety of beneficial, agriculturally significant bacteria, which has resulted in increased nutrient absorption and plant health [235]. PGPR are also important in improving soil fertility, plant health, and pollutant remediation [236]. However, engineering PGPR to produce outcomes in novel agricultural endeavors would be extremely beneficial to the microorganism inoculant industry [229]. One of the extremely important sectors in undeveloped and developed countries is agriculture. The pervasive use of chemicals in modern agriculture has been a source of public concern for the past three decades due to potential negative impacts on the environment, as well as on animal and human health [231]. Agricultural activity is shifting to more sustainable and environmentally responsible strategies around the world, fueled by increasing awareness of the environmental harm and human health risks caused by toxic waste and the overuse of fertilizers and pesticides, prompting increasing demands to address these issues. Therefore, using PGPR to improve plant growth under normal conditions, abiotic stress, and plant pathogen attack is crucial, as these outcomes may not be achieved without PGPR (Figure 2). 

## 7. Mechanisms of Action of PGPR in Bioremediation

### 7.1. Siderophores and Heavy Metal Removal

Microorganisms produce siderophores, which demonstrate good affinity in chelating iron [304,305]. Siderophores are produced by various plant growth-promoting rhizobacteria (PGPR). The solubility of iron phosphate in soil is influenced by siderophores [306]. Due to the formation of siderophores, which are low-molecular-weight proteins, bacterial activities that enhance mineral nutrient absorption by plants can help plants grow in trace metal-contaminated soils [301,307]. Fungi, bacteria, and plants contain iron-chelating secondary metabolites in iron-limiting environments [305,308]. Plant growth can be stimulated by siderophore-producing PGPR either directly by enhancing plant iron nutrition or indirectly by reducing the activity of plant pathogens in the soil root zone by minimizing their iron supply [309]. Iron is more often found as Fe^3+^, which is bound to insoluble oxyhydroxides and hydroxides, making it inaccessible to microorganisms and plants. Fe^3+^ has a greater affinity for siderophores as compared with Fe^2+^ or other trace metals, i.e., Co, Cu, Cd, Pb, Ni, Cr, and Zn [235,301]. As rhizobacterial siderophores tend to reduce the stress exerted by trace metal pollutants, iron supply to developing plants under trace metal stress becomes more critical [310]. Interestingly, phytosiderophores have a lower affinity for iron than bacterial siderophores but require lower iron content for optimal growth as compared to bacteria [306,311]. Plants belonging to the *Poaceae* family have significant potential to enhance the iron supply and its uptake in roots [268,311,312]. Growing siderophore-producing grass species in conjunction with accumulator plants will enhance phytoextraction processes [313]. While siderophore production for the remediation of trace metals has significant potential, phytosiderophores gain their specificity by absorption of iron phytosiderophores via a membrane carrier, rather than by directly chelating iron in soils [235]. Phytoextraction is enhanced by improving the nutrition of plants and mobilizing metals [295]. By selectively promoting iron uptake from the reservoir of trace metal cations vying for transport, siderophore-producing bacteria have been shown to enhance the growth, chlorophyll contents, and biomass of various crops cultivated in trace metal-contaminated soils [306]. Furthermore, the complexation of trace metals by rhizobacterial siderophores in the rhizosphere possibly inhibits the formation of oxidative stress and free radicals [261]. Membrane receptor proteins recognize and scavenge the Fe^3+^ siderophore complexes from the rhizosphere, which are then secreted. They are too large to pass through membrane porins [268]. Instead, ATP-binding cassette transporters mediate transport through the cytoplasmatic membrane, especially in Gram-negative bacteria, and the TonB-dependent transporter proteins transport metabolic energy from the cytoplasm to the outer membrane [305]. However, this type of mechanism is scarcely heard of in Gram-positive bacteria, as they are TonB-dependent transporter proteins [163]. Often, bacteria develop numerous types of siderophores, but the formation of siderophores and various molecules in a contaminated rhizosphere depends on the circumstances, i.e., bacterial strain, soil type, climatic conditions, and pollutant concentration [235,301].

### 7.2. Biosurfactants in Heavy Metal Removal

Biosurfactants classified as amphiphilic molecules, which have large hydrophobic and hydrophilic groups, are one of the most important agents in the remediation of contaminants in the rhizosphere [164,235]. Surfactants have a hydrophilic component (functional groups) that makes them soluble in water and a hydrophobic part that enables them to form hydrophobic interiors and interact with hydrophobic compounds and adsorb at interfaces (e.g., air/water), thereby lowering the surface tension [314,315]. Biosurfactants, the biobased substitutes of surfactants, have risen to prominence as a result of strong toxic manifestations [316]. Biosurfactants, which are microbial surface-active metabolites, are metal-complexing agents that have been shown to be useful in the remediation of heavy metal-contaminated areas [164,304,317]. Biosurfactants have unique properties that make them a possible replacement for conventional remediation methods [314]. These are less toxic in nature and have higher biodegradability and environmental compatibility [318]. Other benefits include their ability to be made from low-cost agro-based raw materials and organic wastes, as well as their ability to maintain functionality even at extreme salt concentration, temperature, and pH in the rhizosphere [319]. The majority of biosurfactants discovered so far come from terrestrial microorganisms [164]. Biosurfactants are produced by a variety of microbial genera, including yeasts, *Enterobacter*, *Rhodococcus*, *Bacillus*, *Halomonas*, *Pseudomonas*, *Arthrobacter*, and *Acinetobacter*. *Brevibacterium* sp. and *Ochrobactrum* sp. are bacteria that produce biosurfactants in crude oil-contaminated soils [237,320]. Emulsifying agents have been identified as *Achromobacter* sp., *Brevibacillus* sp., *Pusillimonas* sp., *Dietzia* sp., and *Sphingopyxis* sp. [321]. In recent studies, it was observed that trace metals, i.e., Cd, Zn, and Pb, had higher affinities for biosurfactants, such as rhamnolipid formed by various *Pseudomonas aeruginosa*, than for many of the soil components to which they are bound in metal-contaminated soils [322,323]. Heavy metal desorption from solid phases is aided by biosurfactants in two ways [164]. The first is the complexation of free metals in solution. According to Le-Chatelier’s principle, this reduces the metal’s solution phase activity and thus promotes desorption [323]. Secondly, biosurfactants aggregate at the solid–liquid interface and decrease the interfacial stress. The authors of [324] also stated that the size and charge of biosurfactants structures influence the flow of biosurfactant–metal complexes through the soil [235]. The ability of biosurfactants to form complexes with metals is central to their bioremediation of trace metal-contaminated soil [325]. Biosurfactant micelles may also strip metal ions from the rhizosphere [236]. The main effectiveness of metal-activated biosurfactant action is influenced by a series of factors, including soil pH, soil particle size, soil structure, cation-exchange capacity (CEC), trace metal concentration, and climatic conditions [164,321]. Because of the long period of contamination, the trace metal has much time to stabilize, making removal more difficult [322]. Biosurfactants’ wide range of applications in organic and trace metal-contaminated soils can be attributed to their low toxicity, small size, biodegradability, cost-effectiveness, and high specificity [326]. The significance of biosurfactants in facilitating systems as biocontrol agents has yet to be fully investigated and warrants further research. Such research will aid in the replacement of harsh chemical surfactants with green alternatives. There is also more work to be carried out regarding the production costs of green surfactants in order to achieve net economic gain from biosurfactant use in the agricultural sector for the remediation of trace metal-contaminated soils.

### 7.3. Biosorption

Biosorption is a simple, metabolically passive physicochemical process that involves the binding of metal ions/biosorbate to the surface of a biologically derived biosorbent [327,328]. Biopolymers, industrial or agriculture waste, plant-derived materials, microorganisms, and other biological removal materials are used for biosorption remediation of trace metals [329]. In contrast to oxidation through anaerobic and aerobic metabolism, it is a reversible, rapid process that involves the binding of ions to the functional groups present on the surface of the biosorbent in aqueous solutions through various interactions [330,331]. Operational simplicity, low quantity of sludge generation, high efficacy, the lack of requirements for additional nutrient or increased chemical oxygen in water, regeneration ability of the biosorbent, and low operational cost are recognized as some of the benefits of this process [332]. Biosorption can eliminate contaminants even at low concentration, which is especially important for the removal of trace metals because they are toxic at ppb levels [333]. For the biosorption process, either living or dead microorganisms or other agricultural and industrial byproducts may be used as biosorbents [293]. In the first step of trace metal ion biosorption, the biosorbent should be suspended in a solution containing the biosorbate [334]. Equilibrium is achieved after a defined period of incubation. The metal-enriched biosorbent will be isolated at this point [332,335]. Biosorption is beneficial since it is reversible, does not require nutrients, has a single-stage mechanism of short duration, poses no risk of harmful effects or cellular expansion, allows for intermediate equilibrium concentrations of trace metal ions, and is not regulated by metabolism [336]. The attachment of the sorbate to the biosorbent is a dynamic process that occurs during biosorption [337,338]. Examples of biosorbent mechanisms to remove trace metal ions include physical (van der Waals forces or electrostatic reaction) or chemical (displacement of bound trace metal cations, ion exchange, or protons) binding, complexation, precipitation, reduction, and chelation [293,328,339]. Biosorbents contain functional/chemical groups that can attract and sequester trace metal ions, such as phosphate, imine, phenolic, phosphodiester, carboxyl, sulfhydryl, carbonyl, sulfonate, thioether, imidazole, amide, and amine [329,335]. The biosorption mechanism is influenced by a number of factors, including soil pH, soil temperature, initial metal ion concentration, biomass concentration, agitation speed of the biosorbent, and biosorbent concentration [340,341]. In terms of trace metal detection, major improvements have been made in the last decade with the use of optical chemical sensors, graphene-modified nanomaterials, and other biomonitoring instruments [342]. Given these technological advances and the ability to reuse metal resources, biosorption is a competitive, affordable, and promising mechanism, and it can foster sustainable economic development in countries. 

### 7.4. ACC Deaminase Activity and Reduction in Ethylene Levels

The trace metal stress-induced acceleration of ethylene development in plants is modulated by rhizobacteria with ACC deaminase activity, and this alteration in root architecture and the plant’s root uptake system can result in increased uptake of inorganic contaminants [15,343]. ACC deaminase rhizobacteria have great potential for the development of bacterial inoculation in a trace metal-contaminated rhizosphere to boost plant growth in unfavorable environments, particularly for hyperaccumulators [155,344]. The ACC deaminase derived from a rhizobacterium (*Kluyvera ascorbata* L. SUD165) has a higher potential to protect tomato (*Lycopersicon esculentum* L.) and canola (*Brassica napus* L.) seeds from nickel chloride toxicity when cultivated under gnotobiotic conditions by reducing the levels of ethylene under Ni stress [300]. Ethylene is considered a stress hormone that has been shown to encourage plant growth at low concentrations, but at moderate to high concentrations, it can prevent root elongation and senescence of the plant [345]. In another study, Belimov et al. [316] observed that rhizobacteria ACC deaminase promoted the growth of roots and shoots in canola seedlings grown in soil contaminated with 300 mM CdCl_2_. A goal of the study was to find a connection between rhizobacteria with in vitro ACC deaminase activity and increased cadmium accumulation in plant tissues due to increased root growth caused by the rhizobacteria [269]. Furthermore, ACC deaminase producing rhizobacteria are used to create plant–inoculant systems for phytoremediation of contaminated soil environments. Similarly, in another experiment, inoculation with a rhizobacterium *Pseudomonas putida* L. 06909 caused a significant decrease in Cd phytotoxicity and increased metal accumulation in sunflower (*Helianthus annus* L.) plant root by 40% [279]. It is highly probable that, in addition to other characteristics, rhizobacterium *Pseudomonas putida* L. 06909 has ACC deaminase activity, which aids in the reduction of trace metal phytotoxicity and increased metal aggregation in plant roots. Li et al. [288] observed that tobacco (*Nicotiana tabacum* L.) plants inoculated with a rhizobacterium (*Pseudomonas putida* L. UW4) showed good development and absorbed a significant amount of nickel from nickel-contaminated soil. The rapid growth of hyperaccumulators induced by ACC deaminase rhizobacteria can aid not only in remediation but also in the uptake of trace metals [15,345]. Using transgenic hyperaccumulator plants that express ACC deaminase is a technique for the phytoremediation of trace metal-contaminated agricultural soils [343]. However, further research is required to investigate different facets of this technique in order to improve its efficacy in the remediation of trace metals. 

### 7.5. Production of Exopolysaccharides and Polymeric Substances

Since the discovery of the bacterial exopolysaccharide (EPS) adsorption potential, numerous studies have been published on a diverse range of bacterial species and EPSs with the required capability, i.e., remediation of trace metal-contaminated soils [346,347]. EPS, which has a simple polysaccharide backbone, can be structurally modified by changing the polymeric length or by adding an array of different side chains, functional units, non-carbohydrate substituents, and various bonds and linkages in a combinatorial manner [348,349]. The nature and percentage of the available carbon source, abiotic stress factors, including pH, temperature, and trace metals, and the growth process of rhizobacterium during which synthesis occurs all play roles in determining the polysaccharide composition [350,351]. The utilization of negatively charged EPS (EPS with abundant anionic functional groups) as a viable biosorbent must be emphasized in strategies for trace metal remediation using rhizobacterial EPSs [352,353]. Abundant ionizable and active non-carbohydrate side chains and functional groups, such as acetamido (chitin group), structural polysaccharides (fungi), sulfhydryl, amine, and carboxyl groups present in proteins and phosphodiester, hydroxyl, and phosphate groups in polysaccharides, impart an overall negative charge to the polymer [354]. In addition, unlike homopolysaccharides, extracellular heteropolysaccharides are frequently polyanionic due to the interaction of some of these functional groups with the polysaccharide backbone [355]. Immobilization and sorption occur by various processes, i.e., complexation, ion exchange, and precipitation. Certain documented commercial rhizobacterial EPS strains having the potential of anionicity are *Enterobacter* sp. A47, *Pseudomonas oleovorans* L., *Xanthomonas campestris* L., *Streptococci* sp., *Pasteurella multocida* L., *Pseudomonas aeruginosa* L., *Sphingomonas paucimobilis* L., and *Azotobacter vinelandii* L. [347,350,351]. The association between positively charged trace metal ions and negatively charged EPS and cell surfaces causes EPS- or intact microbial cell (dead or alive)-mediated biosorption [356]. *Herminiimonasarsenicoxydans* L., a rhizobacterium with a Gram-negative phenotype, has been reported not only to cause or activate the formation of biofilm in response to arsenic (As) contamination but also to use EPS to scavenge arsenic ions when exposed to concentrations up to 5 mM [351,357]. This study shows that although the synthesis of rhizobacterial EPS may not be induced in response to trace metal stress, the formed EPS can also adsorb the metal. Similarly, EPSs produced by *Marinobacter* sp. have been evaluated for the remediation of trace copper (Cu) and lead (Pb) [358]. However, a variety of safer rhizobacteria, which are scattered in the environment, are waiting to be discovered for the remediation of environments contaminated with trace metals. These EPS producers should be investigated for trace metal ion chelation capabilities, as they can develop a potent polysaccharide with anionic moieties.

## 8. Pros and Cons of Rhizosphere Bacteria for Agricultural Sustainability

**Figure 3:**
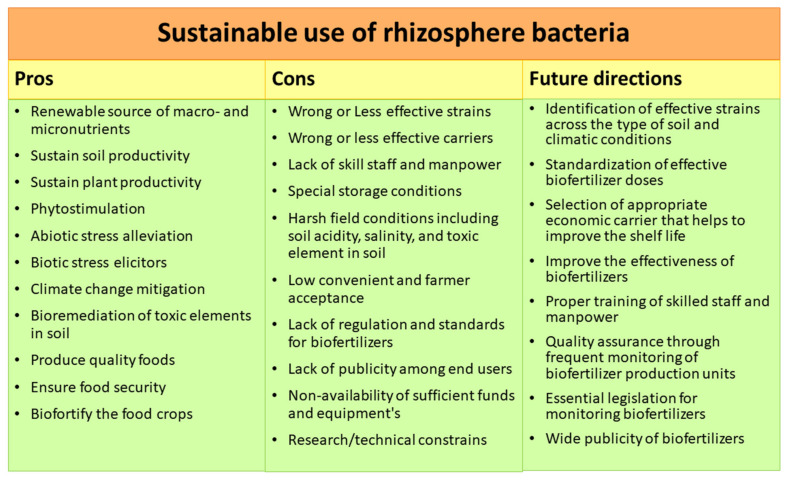
Comparison of pros and cons of rhizobacterial application for sustainable agriculture with possible future directions [359,360,371,372,373,374].

Rhizosphere bacterial inoculants are indisputably necessary for the augmentation of plant growth and maintenance of soil output. As reported in the above sections, rhizosphere bacterial inoculants benefit plants through various mechanisms, although some studies indicate adverse effects [5]. In this section, we compare the pros and cons of rhizosphere bacterial biofertilizers, and a comparison of such biofertilizers is presented in Table 5 and demonstrated in Figure 3.

### 8.1. Pros of Rhizosphere Bacterial Application

Numerous commercially available microbial biofertilizers are sold as dried or liquid cultures under a variety of trade names, as listed in Table 5. The application of such rhizosphere bacterial biofertilizers could have an impact on agricultural sustainability and phytopathogen biocontrol and sustain soil and plant production by improving nutrient availability and reducing the application of chemical fertilizers and pesticides. The bioremediation and biodegradation of hazardous substances of biological or anthropogenic origin could also be improved [375]. Various beneficial rhizosphere bacterial inoculants could be formulated in the form of products, including biofertilizers. Such products are viable sources of nutrients that could act as alternatives to chemical fertilizers, stimulate plant growth, remediate heavy metal-contaminated environments, and mitigate environmental stresses [376,377]. 

The application of rhizosphere bacterial biofertilizers has many advantages over the conventional fertilization system. Initially, a small amount of rhizosphere bacterial inoculum is sufficient for the preparation of biofertilizer. Their application does not require energy sources for their survival under field conditions, as they possess saprophytic nutritional requirements that make their large-scale application feasible [378]. Their application is simple in terms of seed, soil, and/or root treatments. Crop seeds are treated with solid or liquid formulations of rhizosphere bacterial inoculants with or without a carrier material. Such formulations may also be directly applied in the field along with compost fertilizers. The inoculated bacteria can survive in the vicinity of the rhizosphere and colonize plant roots, where they have a beneficial influence on plants [379]. These bacterial inoculants spread along with the root system and improve plant growth through their versatile metabolism, as briefly discussed in the above sections. 

The application of such bacterial biofertilizers sustains soil health and productivity by improving the soil bacterial community structure and composition and affecting afforestation [380]. Such biofertilizers benefit plants by facilitating renewable nutrient availability via atmospheric N fixation and solubilization of nutrients. The three types of N-fixing biofertilizers, namely, free-living, symbiotic/endophytic, and rhizosphere bacteria, were reported to enhance N availability to crops [381]. For example, rhizobia biofertilizers can fix 50–300 kg N ha^−1^, increase yield by 10–35%, protect soil fertility, and improve residual N availability for subsequent crops [382]. Various nutrient-solubilizing rhizosphere bacterial biofertilizers were also reported to increase the availability, uptake, translocation, and accumulation of P, K, Zn, Fe, Se, Mn, and Si by solubilizing them from minerals and producing various organic and inorganic acids [383]. Such biofertilizers are nonspecific and can be used on any crop. They produce enzymes to convert insoluble organic P to a soluble form and have increased crop yield by 10–30% [68]. These nutrient-solubilizing rhizosphere bacteria improve nutrient uptake, accumulation, and translocation into cereal grains and could be a promising option for the biofortification of cereals [80]. Rhizosphere bacterial inoculants mediate photostimulation by communicating with plants via quorum sensing, which enables bacteria to synchronize the expression of genes and behavior. Bacterial inoculants produce N-acyl-L-homoserine lactones, cyclodipeptides, and various phytohormones that are involved in the stimulation of plant growth and defense [384]. The application of rhizosphere bacterial inoculants could modulate systemic mechanisms in a plant to enhance its defense against adverse abiotic and biotic stress conditions. Resistance to biotic stress is elevated through the production of secondary compounds called allelochemicals that induce immunity against pathogen attack. Additionally, such bioinoculants play a multifaceted role in the alleviation of abiotic stress due to climate alterations and in the restoration of natural soil against a variety of toxic metals [87]. 

### 8.2. Cons of Rhizosphere Bacterial Application

Recently, there has been growing interest in the use of biofertilizer products. However, their application faces serious constraints at various levels, from laboratory screening to field applications. We classify all biofertilizer constraints broadly into research and development, regulatory and marketing, and field-level constraints. Such constraints are described in detail in the following subsections. 

#### 8.2.1. Research and Development Constraints

The biofertilizer constraints encountered in research and development can be classified into biological, technical, quality control, carrier, and biosafety constraints. Among the biological constraints, we may often fail to select a potential PGP rhizosphere bacterial strain, as this is a difficult task. For biofertilizer development, the initial evaluation of rhizosphere bacterial isolates for PGP characteristics under laboratory conditions does not ensure their efficacy in field conditions. For example, isolates from pure cultures demonstrating less in vitro growth-promoting activity showed greater plant growth promotion under field conditions. The potential isolated strains occasionally pose a challenge to screening due to the incomplete understanding of potential PGP mechanisms. As a result, such potentially useful strains with PGP mechanisms are occasionally rejected owing to their unsatisfactory results during in vitro experiments [371]. The efficiency of applied inoculants deteriorated in the presence of many other microorganisms under field conditions. Thus, target strains should be chosen based on their performance under field conditions, and they should be applied to various crops and grown in a variety of soil types [385]. The selected bioinoculant in a biofertilizer could be less effective at replacing native ineffective strains, less compatible with a crop, and less capable of colonizing host plant roots and thriving in soil [367]. 

The shelf life of biofertilizers and the commercialization of a successful rhizosphere bacterial inoculant remain as major challenges [386]. Biofertilizers with a short shelf life need to be recycled before expiration, which results in financial losses for the associated firm. Their storage and transportation require additional caution because biofertilizers are composed of live bacterial cells and can deteriorate under harsh environmental conditions [367]. A mutation in a bioinoculant may create a serious problem if it decreases its efficiency and thus raises the cost of production. To increase the shelf life of biofertilizers, a suitable carrier is needed for field application. The lack of a suitable carrier is a significant restriction on its widespread usage in fields. Peat, charcoal, and lignite are considered excellent carriers for biofertilizer processing; however, the majority are in short supply in developing countries, and mining of these carriers has been downscaled in developed countries. A potential carrier should be inexpensive, nearly sterile, and free from moisture and toxic substances in addition to having both high organic matter content and water-holding capacity [372]. At the moment, there are no quality control procedures for biofertilizers. It is necessary to develop quality control standards for biofertilizers to demonstrate their efficacy in promoting plant growth on a field scale [367]. 

#### 8.2.2. Regulatory and Marketing Cnstraints

Biofertilizer production and quality control require advanced technology and highly qualified and trained human resources. The major infrastructure limitations are a lack of advanced technology, necessary technical support and equipment, trained workforce, and skilled technical personnel [367]. Regulatory constraints include difficulties in registering biofertilizer products and filing patents due to frequently changing and inconsistent regulations among regions and countries. The entire regulatory process of developing a potential biofertilizer, from registration to commercialization, is lengthy, potentially taking several years, and quite complex [385]. Financial constraints are a serious hindrance to large-scale biofertilizer production. After manufacturing the biofertilizer, small producers lack the financial resources necessary to distribute it independently. As a result of a delay in its distribution, the product quality deteriorates, which reduces its potential [367]. The lack of adequate transportation and storage facilities is a significant impediment to commercialization. Farmers either lack or have insufficient knowledge about the sustainable agriculture benefits of biofertilizers over hazardous agrochemicals. As a result, demand for such green products declines. Due to a shortage of qualified technical personnel, the establishment of extension centers has little effect on increasing farmer awareness [367]. During packing and marketing, the rhizosphere bacterial bioinoculants are exposed to high temperatures (≥40 °C), which may result in their inactivation or death, thereby reducing their value as biofertilizers. As a result, these low-quality packets will be detrimental to farmers and efforts to increase the overall crop yield. 

#### 8.2.3. Field–Level Constraints

Farmers are skeptical of biofertilizers due to the extremely slow and frequently unsuccessful crop responses to applied biofertilizers, as the inoculum requires a longer time to colonize the roots and for the effective concentration to be established. Biofertilizer efficacy is reduced in the field due to the residual properties of harmful chemicals [367]. Environmental stresses contribute significantly to the reduction in biological activity in some areas. Several other factors contributing to the poor performance of biofertilizers include nutrient availability, soil acidity and alkalinity, high and low temperatures, pesticide application, radiation, and high nitrate concentrations in the soil, which limit the bioinoculants’ ability to fix atmospheric N, solubilize nutrients, and interact with indigenous soil microbiota, which influences the presence and survivability of rhizosphere bacteria and the host plant [387]. Numerous soils are contaminated with heavy metals as well as deficient in other critical nutrients, which reduce the biological potential of rhizosphere bacterial inoculants in biofertilizers [388]. Region-specific rhizosphere bacterial biofertilizers should be identified to optimize the effectiveness of the used strains. Soil fumigation with broad-spectrum biocidal fumigants has a deleterious effect on the soil microbial community [389]. The inconsistent application of biofertilizer limits the presence of viable bacterial populations, which results in their inefficiency in promoting the growth of agronomic crops. Typically, farmers expect rapid, visible outcomes from a single application of biofertilizers, which represents another serious limitation to their wide-scale application. The limited application of biofertilizer could be due to the lack of awareness in farmers about the concentration, time, and method of biofertilizer application. Repeated applications of biofertilizer are needed to maintain the bacterial numbers and ensure a viable PGP rhizobacterial population in soil for alleviating various environmental stresses. However, the literature is limited in specifying the required biofertilizer dosage. Therefore, extensive research is required to evaluate the optimal dose of biofertilizers and their effects on crop productivity and stress alleviation.

## 9. Future Directions 

The application of rhizosphere bacterial strains represents a sustainable technology in agriculture being embraced in many developed countries. Native plant microbiomes play an important role in increasing plant survival under abiotic and biotic stresses [390]. To promote crop productivity, the desired plant growth-promoting traits could be inserted into rhizosphere bacterial strains through recent innovative genetic engineering technologies. It is difficult to screen the bulk of rhizosphere bacterial strains to identify advantageous traits under stress conditions; however, a limited number of beneficial bacterial strains can be isolated, engineered, and applied to crops [373]. The importance of identifying and evaluating novel beneficial rhizosphere bacterial strains is emphasized. Core plant microbiomes can be attained through screening and signal transmission during various growth stages, including seed transfer and plant germination [391]. The intrinsic plant ability to stimulate the rhizosphere bacterial community can be useful in selecting the desirable strains. Rhizosphere bacterial strains can be subjected to whole-genome sequencing to differentiate their functional capabilities. Native plant rhizosphere bacterial diversity can be in situ modified through prevailing biochemical and molecular-based approaches [374]. Collective efforts are needed to realize the potential impact of rhizosphere bacteria on environmental restoration by broadening their applications. Public misunderstanding and misinformation must be overcome by educating the public about the advantages of rhizosphere bacteria. Novel strategies must be explored for the formulation and transfer of rhizosphere bacterial biofertilizer at the laboratory, greenhouse, field, and market scales to sustain agricultural productivity and the environment. The laboratory support of detailed mechanistic studies on the ability to stimulate growth and bioremediate environments contaminated with pollutants is essential. More mechanistically effective strains might be genetically engineered and regulated according to approved policies to prevent future hazards. 

## Figures and Tables

**Table 1:** Summary of studies on rhizobacterial mechanisms involved in plant growth promotion.

PGPR Candidate	Host Plant	Growth Condition	Proposed Mechanism(s)	Plant Response	References
*Rhizobium tropici*	Bean	Pot	N_2_ fixation and IAA production	Increase in number of nodules and plant dry weight	[45]
*Pseudomonas stutzeri* A1501	Maize	Field	N_2_ fixation and ACC deaminase production	Better growth and more nitrogen accumulation	[46]
*Rhizobium etli*	Bean	Pot	Nitrogenase activity	A 16% increase in the N_2_ content of the resultant seeds and a 25–30% increase in leaf nitrogen	[47]
*Pseudomonas fluorescens* BSP53	Blackcurrant cuttings	Pot and field	IAA overproduction	Root development	[45]
*Streptomyces* sp. NEAU-S7GS2	Soybean	Pot	IAA production and inorganic phosphate solubilization	A 77% increase in plant growth and a 38% decrease in sclerotinia stem rot of soybean	[48]
*Halobacillus dabanensis strain* SB-26 and *Halobacillus* sp. GSP 34	Rice	Pot	IAA production and nitrogen fixation	Enhanced plant height and yield	[49]
*Fluorescent Pseudomonas* sp. PF17	Sunflower	Pot	IAA production and phosphate solubilization	Promoted overall plant growth	[50]
*w*ild-type *P. putida* GR12-2	Mung bean	Pot	IAA-overproducing and pH-lowering mechanism	Root and shoot development	[51]
*R. leguminosarum* bv. *Viciae*	Alfalfa	Pot	ACC deaminase	Increase in nodule number and biomass	[44]
*Bacillus* sp. HWP47	Wheat	Pot	ACC deaminase activity and chelation mechanism	Caused 51.46% increase in root dry weight (RDW)	[52]
*Rhizobacteria* sp.	*Solanum tuberosum*	Pot	ACC deaminase activity and phytohormone production	ROS antioxidant enzyme expression and improved photosynthesis	[53]
*Pseudomonads*	Tobacco	Field	Ferric siderophore production	Adventitious root development	[54]
*Pseudomonas fluorescens C7*	*Arabidopsis thaliana*	Pot and Field Trials	Fe-pyoverdine complex	Increase in iron inside plant tissues and hence enhanced plant growth	[55]
*Bacillus mucilaginosus* HQ013329	Garlic	Pot	Various extracellular polymers are produced (primarily proteins and polysaccharides)	Promoted garlic plant growth characteristics and avoided environmental pollution hazards caused by heavy application of potassium fertilizers	[56]
*Bacillus circulans*	Pepper Potato	Pot	Acidolysis of the surrounding area of microorganism	Highest total yield of pepper and potato tuber yield per plant and average tuber weight	[57]
*B. mucilaginosus* strain RCBC13	Tomato	Pot	Production of α-ketogluconic acid	125% increase in biomass, whereas K and P uptake were more than 150% in tomato	[58]
*Pseudomonas aeruginosa* PF_23_^EPS+^	Sunflower	Pot and field	Salicylic acid production	Improved growth of crop in salinized soil	[59]
*Bradyrhizobium japonicum* USDA 110 with salt-tolerant *Pseudomonas putida* TSAU1	Soybean	Hydroponic	Formation of nodules	Increased plant growth and nitrogen and phosphorus uptake	[60]
*Bacillus amyloliquefaciens* subsp. *plantarum* UCMB5113	*Arabidopsis thaliana*	Lab	Improved root growth and configuration	Increased plant growth and reduced disease effect	[61]
*Bacillus firmus* SW5	Soybean	Pot	Improved root structure	Increased biomass, nutrient uptake, and gas exchange parameters	[62]
*Azospirillum brasilense*	Maize	Pot and field	Siderophore production	Increased biomass and improved plant health	[63]
*Acidovorax delafieldii*	Rice	Pot	Siderophore production and tricalcium phosphate solubilization	Enhanced biomass, grain yield, and NPK uptake	[64]
*Brevibacillus brevis* GZDF3	*Pinellia ternata* (an important herb in traditional Chinese medicine)	Lab	Siderophore production	Helpful in the development of new biological agent	[65]
*Bacillus subtilis* MF497646 and *Pseudomonas koreensis* MG209738	Maize	Pot and field	Siderophore production	Increased antioxidant activities and yield parameters	[66]

**Table 2:** Summary of studies on rhizobacterial mechanisms involved in biocontrol.

Biocontrol Agent	Plant Pathogen	Host Plant	Proposed Mechanism(s)	Reference
*Pseudomonas fluorescens*	*Fusarium culmorum*	Rye	Fe(III)-chelating compounds (including siderophores)	[159]
*Acinetobacter, Pseudomonas, Staphylococcus, Bacillus, Enterobacter, Pantoea, Alcaligenes*	*Fusarium oxysporum*, *Alternaria alternate*, *F. culmorum*, *F. solani*, *Botrytis cinerea*, *Pythium ultimum*, *Phytophthora cryptogea*	Wheat	Antagonism and growth promotion	[155]
*Bacillus* sp. L324-92	*Gaeumannomyces graminis* var tritici, *Rhizoctonia* root rot, *R. solani* AG8, *Pythium* root rot, *Pythium irregulare P. ultimum*.	Wheat	Not specified	[90]
*Bacillus* sp., *Pseudomonas fluorescens*	*R. oryzae, P. ultimum, G. graminis, R. solani*	Wheat	Not specified	[160]
*Pseudomonas fluorescens*	*Microconidium nivale*/ *Fusarium nivale*	Wheat	Growth promotion, siderophore production, in vitro antibiosis	[161]
*Bacillus subtilis* and *B. cereus*	Take all (*G. graminis var tritici*) Rhizoctonia root rot (*R. solani*AG8)	Wheat	Growth promotion	[162]
*Bacillus subtilis* CE1	*Fusarium verticillioides*	Maize	Not specified	[163]
*Pseudomonas chlororaphis*	*Macrophomina phaseolina* (charcoal rot of sorghum)	Sorghum	Extracellular antibiotics, production of volatiles, siderophores, effective root colonization	[164]
*Pseudomonas fluorescens* MKB 100 and MKB 249, *P*. *frederiksbergensis* 202, *Pseudomonas* spp. MKB 158	*Fusarium culmorum*	Wheat and barley	Induced resistance, antibiotic production, pathogenesis-related proteins (induced resistance) in wheat	[165]

**Table 3:** Summary of studies on the effects of rhizobacterial strains involved in the bioremediation of polyaromatic hydrocarbon-contaminated soils.

PGPR Candidate	Pollutants	Pollutant Concentration	Remediation Efficiency (%)	References
*Azoarcus* sp., *Escherichia coli* L.	Benzoates	1 mM	90.0	[248]
*Pseudomonas fluorescens* L. and *Pseudomonas putida* L.	Benzoates and related substances	1 mM	60.0	[227]
*Rhizobium leguminosarum* L.	Chrysene	500 mg kg^-1^	28.0	[249]
*Azospirillum lipoferum* L. and *Azospirillum brasilense* L.	Crude oil	1% v/v	57.0	[228]
*Acinetobacter lwoffii* L., *Bacillus subtilis* L., and *Pseudomonas aeruginosa* L.	Crude oil	1% v/v	89.0	[250]
*Alcaligenes faecalis* L., *Citrobacter murliniae* L., *Dietzia papillomatosis* L., *Nocardioides deserti* L.	Crude oil	1174 mg/L	90.0	[251]
*Enterobacter* sp. MN17	Crude oil	5000 mg kg^−1^	63.0	[229]
*Enterobacter cloacae*	Crude oil	2000 ppm	54.0	[230,231]
*Pseudomonas cepacia* L. and *Arthrobacter* sp.	2,4-Dichlorphenoxyessigsäure (kurz 2,4-D)	5% v/v	80.0	[236,252]
*Rhizopus arrhizus* L. and *Pseudomonas aeruginosa* L.	Dichlorodiphenyltrichloroethane (DDT)	1 mg L^−1^	34.0	[237,253]
*Chroococcus* sp. and *Synechocystis* sp.	Linurin	1 mg L^−1^	>98	[254]
*Pseudomonas* sp., *Pseudomonas aeruginosa* L., *Pseudomonas stutzeri* L.	Parathion	3800 µg/mL	>49	[255]
*Archaea* sp., *Acinetobacter* sp., *Achromobacter* sp., *Alcaligenes* sp., *Aspergillus* sp., *Arthrobacter* sp., *Azotobacter* sp., *Bacillus cereus* sp., *Flavobacterium* sp., *Neurospora* sp.	Phenolic compounds	13.0 mg L^−1^	99.0	[256,257]
*Leptolyngby*a sp., *Penicillium* sp., *Candida tropicalis* L., *Debaryomyces subglobosus* L., *Pseudomonas aeruginosa* L., *Pseudomonas putida* L., and *Trichosporon cutaneum* L.	Phenolic compounds	150.0 mg L^−1^	99.0	[258]
*Pseudomonas* sp.	Phenanthrene	200 mg kg^-1^	>62	[259]
*Actinobacteria* sp.	Phenanthrene	100 mg L^−1^	50.0	[260]
*Pseudomonas putida* L.	Phenanthrene	100 mg kg^-1^	89.0	[261]
*Pseudomonas* sp.	Phenanthrene	100 mg kg^−1^	73.0	[228]
*Pseudomonas* sp.	Phenanthrene	100 mg kg^-1^	50.0	[239]
*Actinobacteria* sp., *Caulobacterales* sp., *Rhizobiales* sp., *Rhodococcus* sp., *Xanthomonadales* sp.	Phenanthrene	1260 mg kg^−1^	48.0	[241]
*Pseudomonas cepacia* L.	2,4,5-Trichlorophenoxyacetic acid (2,4,5-T)	200 µM	27.0	[262]
*Alcaligene* sp., *Bacillus* sp., *Citrobacter* sp., *Corynebacterium* sp., *Flavobacterium* sp., and *Pseudomonas* sp.	Surfactants	200 mg L^−1^	50.0	[263]
*Bacillus* sp., *Candia* sp., *Pseudomonas* sp.	Surfactants	300 mg L^-1^		[264]
*Burkholderia multivorans* L.	Surfactants	150 mg L^-1^	41.0	[265]
*Pseudomonas* sp.	Surfactants	2% v/v	94.0	[266]

**Table 4:** Summary of studies on rhizobacterial mechanisms involved in the bioremediation of heavy metal-contaminated soils.

Heavy Metals	Microbes	Host Plant(s)	Proposed Mechanism(s)	References
As	*Penicillium aculeatum* PDR-4	Sunflower	Phosphatase, siderophore	[267]
Cd	*Bacillus* sp., *Endophytic* sp., *Rahnella* sp., *Enterobacter* sp., *Klebsiella* spp., *Pseudomonas* sp., *Arthrobacter* sp. TISTR 2220, *Agromyces* AR33, *Brevundimonas* Kro13, *Ralstonia* sp. TISTR 2219, *Streptomyces* AR17, *Variovorax paradoxus* L.	Wheat, Mustard, Soybean, Mung bean, Willow, Amaranthus, Sunflower, Basil, Goat willow	Exopolysaccharide production, siderophores	[231,268,269,270,271,272,273,274]
Cd	*Bacillus badius* L., *Cronobacter muytjensii* L., *Drepanomonas revolute* L., Euplotes sp., *Pseudomonas azotoformans* L., *Uronema nigricans* L.	–	Biosorption	[275,276,277,278]
Cd	*Alcaligenes* sp., *Bacillus* sp., *P*seudomonas sp., *Rhodococcus* sp., and *Variovorax* sp.	Canola, Sunflower	ACC deaminase activity and reduction in ethylene levels	[68,279]
Co	*Enterobacter ludwigii* L.	Sunflower	Exopolysaccharide production	[280]
Cr	*Aerococcus* sp., *Bacillus* sp., Endophytic sp., *Rhizobacteria* sp., *Staphylococcus* sp., *Ochrobactrum intermedium* L., *Pseudomonas aeruginosa* L., *Pseudomonas* *pseudoalcaligenes* L.	Chickpea, Mesquite, Prosopis, Maize, Sorghum, Wheat, Mung bean, Soybean, Sunflower	Exopolysaccharide production	[271,281,282,283,284,285]
Cr	*Bacillus circulans* L., *Bacillus megaterium* L., *Cronobacter muytjensii* L., *Saccharomyces cerevisiae* L.	–	Biosorption	[276,286,287]
Cu	*Pseudomonas* sp., *Rhizobacteria* sp., *Bacillus subtilis* L., *Brevibacterium halotolerans* L., *Bacillus* *pumilus* L., *Brevibacterium casei* MH8a, *Paenibacillus polymyxa* L., *Pseudomonas pseudoalcaligenes* L.	Maize, Sunflower, Sorghum, Mustard, Trailing daisy	Exopolysaccharide production, siderophores	[282,288,289,290,291,292]
Cu	*Aspergillus flavus* L., *Cronobacter muytjensii* L., *Pseudomonas azotoformans* L., *Drepanomonas revolute* L., *Euplotes* sp., *Uronema nigricans* L., *Pichia guilliermondii* L.	Maize, Wheat, Sorghum	Biosorption	[275,276,277,293,294]
Fe	*Pseudomonas* sp., *Psychrobacter* sp.	Castor bean	Exopolysaccharide production, siderophores	[295]
Ni	*Micrococcus* sp., *Pseudomonas* sp., *Psychrobacter* sp., *Bacillus subtilis* SJ-101, *Bacillus pumilus* L., *Rhodococcus erythropolis* X79289, *Rhodococcus globerulus* X80619	Mustard, *Alyssum* sp., Alpine pennycress, Castor bean, Mung bean, Wheat, Soybean	Exopolysaccharide production, siderophores	[271,295,296,297,298,299]
Ni	*Kluyvera ascorbate* L. (SUD165), *Pseudomonas putida* L. (UW4)	Tomato, Canola, Tobacco	ACC deaminase activity and reduction in ethylene levels	[288,300]
Pb	*Enterobacter* sp., *Klebsiella* sp., *Enterobacter ludwigii* L., *Agromyces*AR33, *Bacillus subtilis* L., *Brevibacterium halotolerans* L., *Bacillus megaterium* HKP-1, *Bacillus* *pumilus* L., *Penicillium aculeatum* PDR-4, *Pseudomonas pseudoalcaligenes* L., *Streptomyces* AR17	Sunflower, Mustard, Maize, Sorghum, Goat willow	Exopolysaccharide production, siderophores	[91,231,272,274, 279,280,282,301]
Pb	*Aspergillus niger* L., *Bacillus xiamenensis* L., *Pseudomonas azotoformans* L.	–	Biosorption	[277,293,302]
Zn	*Enterobacter* sp., *Klebsiella* sp., *Pseudomonas* sp., *Rahnella* sp., *Rhizobacteria* sp., *Psychrobacter* sp. *Agromyces* AR33, *Bacillus megaterium* HKP-1, *Bacillus subtilis* L., *Brevibacterium halotolerans* L., *Brevibacteriumcasei*MH8a, *Bacilluspumilus* L., *Enterobacter ludwigii* L., *Streptomyces* AR17	Mustard, Sunflower, Castor bean, Maize, Sorghum, Goat willow,	Exopolysaccharide production, siderophores	[272,279,280,282,291,295,303]
Zn	*Cronobacter muytjensii* L., *Drepanomonas revolute* L., *Euplotes s*p., *Uronema nigricans* L.	–	Biosorption	[275,276]

**Table 5:** Pros of biofertilizers and their composition.

Pro’s Types	Product (Bacterial Composition)	References
Nitrogen fixers	AgriLife NitroFix (*A. chroococcum*, *A. vinelandii*, *A. diazotrophicus*, A. lipoferum, *R. japonicum*), Ajay *Azospirillum* (*Azospirillum* sp.), Azofer (*A. brasilense*), Azo-N (*A. brasilense* + *A. lipoferum*), Azo-N Plus (*A. brasilense* + A. lipoferum + A. chroococcum), Azoter (*A. chroococcum*, *A. brasilense*, *B. megaterium*), Azotobacterin (*A. brasilense* B-4485), BactoFil A10 (*B. Megaterium* + *A. brasilense*, *A. vinelandii*), BactoFil Soya (*B. japonicum*), BiAgro 10 (*B. japonicum*), Bioboots (*Bradyrhizobium* sp. + *D. acidovorans*), Biofix (*Rhizobia*), BioGro (*C. freundii*, *K. pneumoniae*, *P. fluorescens*), Bio-N (Azospirillum spp.), Cell-Tech (*Rhizobia*), Custom N_2_ (*P. polymyxa*), Dimargon (*A. chroococcum*), Legume Fix (*B. japonicum* + *Rhizobium* sp.), Mamezo (*Rhizobia*), Nitragin Gold (*Rhizobia*), Nitrasec (*Rhizobium* sp.), Nitrofix (*Azospirillum* sp.), Nodulator (*B. Japonicum*), Nodulator PRO (*B. subtilis* + *B. Japonicum*), Nodulest 10 (*B. japonicum*), Nodumax (*Bradyrhizobium* spp.), Phylazonit M (*A. chroococcum* + *B. megaterium*), Rhizofer (*R. etli*), Rhizosum Aqua (Azospirillum sp.), Rhizosum N (A. vinelandii + *R. irregularis*), Rizo-Liq (*Bradyrhizobium* sp., + *M. ciceri*, + Rhizobium spp.), Rizo-Liq Top (*B. japonicum*), Symbion N (*Azospirillum* sp. + *Rhizobium* sp. + *Acetobacter* sp. + *Azotobacter* sp.), TagTeam (*P. bilaii* + *Rhizobia*), TwinN and TripleN (*Azorhizobium* sp. + *Azoarcus* sp. + *Azospirillum* sp.), Zadspirillum (*A. brasilense*),	[11,359,360,361,362,363,364,365,366]
Nutrient solubilizers	Bio Phos (*B. megaterium*), Biozink (PGPR consortia), CataPult (*Bacillus* spp. + *G. intraradices*), CBF (*B. mucilaginosus*, + *B. subtilis*), Fosforina (*P. Fluorescens*), K Sol B (*F. aurantia*), P Sol B (*P. striata*, + *B. polymyxa*, *B. megaterium*), Phosphobacterin (*B. megaterium*), Rhizosum K (*F. aurantia*), Rhizosum PK (*B. megaterium*, + *F. aurantia*, + *R. irregularis*), Symbion van Plus (*B. megaterium*), Zn Sol B (*T. thiooxidans*),	[360,362,363,366,367]
Biopesticides	Biobit, Dipel, and Delfin (*Bacillus thuringiensis* var. kurstaki), Certan (*Bacillus thuringiensis* var. aizawai), Acrobe, Skectal, Vectobac (*Bacillus thuringiensis* var. israelensis), Trident, Novodor (*Bacillus thuringiensis* var. tenebrionis), Ciba-Foil, Agree, Cutlass (*Bacillus thuringiensis* var. conjugates), MVP, M-Trak (*Pseudomonas fluorescens* (Bt toxin), Doom (*Bacillus papilliae*), Invade (*Serratia entomophila*)	[368,369,370]
Other biofertilizers	Amase (*P. azotoformans*), Bioativo (PGPR consortia), EVL Coating (PGPR consortia), Biotilis (*B. subtilis*), Cedomon (*P. chlororaphis*), Cedress (*P. chlororaphis*)	[360,362]

## Data Availability

Not applicable.
